# EATG training academy STEP-UP: skills training to empower patients

**DOI:** 10.7448/IAS.17.4.19593

**Published:** 2014-11-02

**Authors:** Oleksandr Martynenko, Damian Kelly, Vanessa Say

**Affiliations:** 1 Training and Capacity Building, The European AIDS Treatment Group, Brussels, Belgium; 2 Training and Capacity Building, The European AIDS Treatment Group, Manchester, UK; 3 Communications, Packer Forbes, London, UK

## Abstract

**Introduction:**

Most existing conventional capacity building and educational programs are currently executed on ad-hoc basis. Such approach no longer responds to the needs and capabilities of patients, supporters and healthcare providers in their engagement with and contribution to response to HIV/AIDS. In contrast, long-term, course-like trainings have considerably broader thematic scope and are conducive to more effective and sustainable learning, exchange of experience and best practices.

**Method:**

Over the period of one year, the Academy trains a cohort of 20 activists (10 from East Europe and Central Asia and 10 from Western and Southern Europe). The Academy goes beyond “treatment only” paradigm. Conceptually, five training modules are grouped under three larger domains: treatment literacy, treatment advocacy and treatment activism, thus covering most of the topics pertinent to the current discourse of HIV and related co-infections. To ensure cascade effect and sustainability of the learning, the trainees are offered participation in pan-European HIV conferences (EACS and HIV Glasgow) and resources for follow-up activities.

**Results:**

The trainees empirically applied the knowledge to the benefits of their communities. In Uzbekistan, a trainee introduced EACS treatment guidelines to fellow medical students and junior doctors. In Armenia and Albania a series of small-scale trainings were held, outreaching to young homeless people who were traditionally excluded from HIV treatment and prevention discourse in the two countries. A trainee from Spain used the materials of the Academy in his work in Mozambique and the Spanish Ministry of Health. Five trainees engaged in a joint European cross-countries project on treatment literacy for young people who are most at risk of infection.

**Conclusions:**

EATG Training Academy is a unique initiative in the WHO Europe region that both trains future treatment activists and addresses treatment literacy, advocacy and advocacy topics. This type of capacity building can respond to existing HIV-related problems more effectively using less limited resources and reaching out to larger communities.

